# Alzheimer's Progenitor Amyloid‐β Targets and Dissolves Microbial Amyloids and Impairs Biofilm Function

**DOI:** 10.1002/advs.202301423

**Published:** 2023-08-18

**Authors:** Syed Aoun Ali, Ka Hang Karen Chung, Helen Forgham, William P. Olsen, Aleksandr Kakinen, Arunpandian Balaji, Daniel E. Otzen, Thomas Paul Davis, Ibrahim Javed

**Affiliations:** ^1^ Australian Institute for Bioengineering and Nanotechnology The University of Queensland Brisbane Qld 4072 Australia; ^2^ Interdisciplinary Nanoscience Center (iNANO) Aarhus University Gustav Wieds Vej 14 Aarhus C 8000 Denmark; ^3^ Sino‐Danish Center (SDC) Eastern Yanqihu Campus University of Chinese Academy of Sciences 380 Huaibeizhuang, Huairou District Beijing 101400 China; ^4^ Institute of Biotechnology HiLIFE University of Helsinki Helsinki 00014 Finland

**Keywords:** Amyloid‐β, biofilms, CsgA, FapC, gut‐brain axis, microbial amyloids

## Abstract

Alzheimer's disease (AD) is a leading form of dementia where the presence of extra‐neuronal plaques of Amyloid‐β (Aβ) is a pathological hallmark. However, Aβ peptide is also observed in the intestinal tissues of AD patients and animal models. In this study, it is reported that Aβ monomers can target and disintegrate microbial amyloids of FapC and CsgA formed by opportunistic gut pathogens, *Pseudomonas aeruginosa* and *Escherichia coli*, explaining a potential role of Aβ in the gut‐brain axis. Employing a zebrafish‐based transparent in vivo system and whole‐mount live‐imaging, Aβ is observed to diffuse into the vasculature and subsequently localize with FapC or CsgA fibrils that were injected into the tail muscles of the fish. FapC aggregates, produced after Aβ treatment (Faβ), present selective toxicity to SH‐SY5Y neuronal cells while the intestinal Caco‐2 cells are shown to phagocytose Faβ in a non‐toxic cellular process. After remodeling by Aβ, microbial fibrils lose their native function of cell adhesion with intestinal Caco‐2 cells and Aβ dissolves and detaches the microbial fibrils already attached to the cell membrane. Taken together, this study strongly indicates an anti‐biofilm role for Aβ monomers that can help aid in the future development of selective anti‐Alzheimer's and anti‐infective medicine.

## Graphical abstract

Aggregation of Amyloid‐β (Aβ) peptide in the brain is a pathological hallmark of Dementia. This study aimes to resolve the anti‐microbial physiology of Aβ that correlates with the infectious etiology of Alzheimer's disease. Results demonstrate that Aβ can disintegrate microbial biofilms and reduce the cellular attachment of opportunistic gut pathogens, suggesting a potential role for Aβ in the gut‐brain axis.

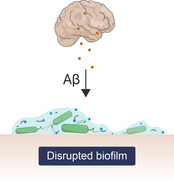

## 1 Introduction

Amyloid‐β (Aβ) is a peptide generated from the proteolytic cleavage of amyloid precursor protein (APP) expressed on the membrane and synapses of neurons. Based on the combination of enzymes that cleave APP, i.e., α‐, β‐ or γ‐secretase, the resulting Aβ can be a 40 (Aβ40) or 42 (Aβ42) residue peptide that is intrinsically disordered in structure.^[^ ^1^ ^]^ Different environmental factors, familial mutations or localized concentrations of Aβ can trigger the aggregation of Aβ into oligomers. The oligomers can subsequently assemble into long twisted fibrils characterized by a so‐called amyloid fold, that is, β‐strands stacked orthogonal to the long fibril axis.^[^ ^2^ ^]^ The abnormal misfolding and aggregation of Aβ drive a major pathological cascade of neuro‐toxicity via membrane poration, resulting in the loss of neurons and progression of Alzheimer's disease.^[^ ^3^ ^]^ As intraneuronal aggregates of Aβ are the characteristic hallmarks of Alzheimer's disease in post‐mortem brain tissues, most major pharmacological efforts have been directed to either suppress the aggregation of Aβ or remove the pre‐formed plaques to treat or at least slow down the clinical progression of the disease.^[^ ^1^, ^4^, ^5^ ^]^ In this context, monoclonal antibodies have made their way into clinical trials. Solanezumab demonstrated some efficacy but not much promise in phase 3 trials against mild form of Alzheimer's disease.^[^ ^6^, ^7^ ^]^ More encouragingly, Lecanemab, a humanized IgG1 monoclonal antibody that binds with soluble protofibrils of Aβ, showed limited improvement in cognition and reduced Aβ burden in phase 2 clinical trials aimed at patients diagnosed with early onset Alzheimer's disease.^[^ ^8^ ^]^

Our understanding regarding the pathology and pharmacology of Aβ oligomers and protofibrils continues to evolve.^[^ ^9^ ^]^ However, the physiological role of Aβ remains unclear. Moving forward with the therapeutic targeting of Aβ, it is important to understand the biological function of Aβ. Additionally, it is necessary that we investigate fully the potential physiological roles of this peptide and examine whether only the aggregated form of Aβ should be removed – making sure we preserve the monomeric peptide for beneficial, intended roles.

Although APP is considered to be significant during synapse formation and neuronal plasticity,^[^ ^10^ ^]^ Aβ was initially considered to be a functionless by‐product of APP catabolism.^[^ ^11^ ^]^ Recently, a new antimicrobial hypothesis for Aβ has begun to emerge^[^ ^12^ ^]^ whereby soluble oligomers of Aβ are thought to bind with the cell‐wall carbohydrate of microbial cells via a heparin‐binding motif in the sequence V_12_HHQKL_17_ of Aβ to induce microbial toxicity.^[^ ^13^ ^]^ Evidence supporting this hypothesis originates from both in vitro and in vivo experiments. For example, four‐week‐old 5xFAD mice that express human Aβ but do not exhibit Aβ plaques and neuroinflammation at this age were able to survive *Salmonella typhimurium* meningitis. This contrasts with APP knockout mice, which succumbed to the infection. Notably, in the 5XFAD mice the endogenous Aβ was colocalized with invading microbial cells present in the brain. Additionally, human brain homogenates taken from Aβ rich regions of human post‐mortem Alzheimer's patient samples exhibit higher antimicrobial activity against *Candida albicans*. Similarly, in Aβ expressing *Caenorhabditis elegans*, Aβ fibrillates into protofibrils and co‐localizes with the invading *Candida* cells inside the gut of the worms.^[^ ^13^ ^]^ Interestingly, Aβ has a well‐established association with the gut where Aβ aggregates can be found in the absorptive epithelial cells of the small intestine.^[^ ^14^ ^]^ Moreover, studies have shown that exogenous Aβ injected into the intestine can propagate, in a prion‐like fashion, to the brain.^[^ ^15^ ^]^

Different microbial pathogens such as *P. aeruginosa* and *Escherichia coli*, which are opportunistic pathogens from the gut or sites of infection, produce fibrillar structures that are analogous to Aβ amyloids.^[^ ^16^ ^]^ Specific proteins, FapC and CsgA are secreted by *P. aeruginosa* and *E. coli*, respectively, and they can self‐assemble into amyloids with a similar mechanism of cross‐β‐sheets stacking.^[^ ^17^, ^18^ ^]^ Notably, CsgA is the major subunit of the curli protein that drives amyloid formation for *E. coli*.^[^ ^19^ ^]^ CsgA curli amyloids are also reported to have a role in Alzheimer's pathogenesis through endocrine activation along the gut‐brain axis^[^ ^20^ ^]^ and increasing HIV virulence by cross‐seeding the fibrilization of prostatic acid phosphatase (PAP248‐286).^[^ ^21^ ^]^ These microbial amyloids provide underlying scaffolds for further deposition of lipopolysaccharides, DNA, and lipoproteins.^[^ ^22^ ^]^ The resultant “nano‐structures” constitute microbial biofilms that encase and protect the microbial colonies against environmental threats and anti‐microbial agents and provide mechanical stability to the ensuing biofilm.^[^ ^23^, ^24^ ^]^

Therefore, considering that Aβ can permeate across BBB through either side; has demonstrated anti‐microbial activity; has been found in the intestinal tissues of the patients and experimental models; and colocalize with the gut microbes, it is reasonable to hypothesize that Aβ peptide has a role in the gut‐brain axis. Motivated by the question of how to determine the native physiological role of Aβ, here we demonstrated that the monomeric form of recombinant human Aβ1‐42, used in this study, can specifically target and dissolve microbial biofilm related amyloids, thereby reducing biofilm attachment with host cells and increasing the susceptibility of the bacterial colony for antibiotics.

## 2 Results

### 2.1 Aβ can Disintegrate/Remodel FapC and CsgA Amyloids of *P. aeruginosa* and *E. coli*

**Figure 1:**
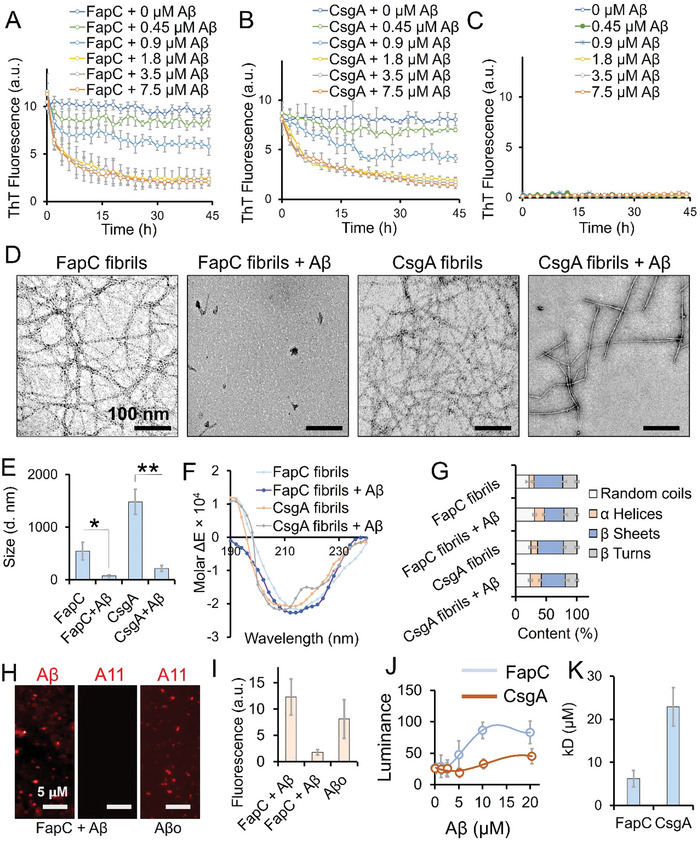
Aβ disintegrate FapC and CsgA amyloids of *P. aeruginosa* and *E. coli*. A–C) ThT assay showing disintegration of 50 µm FapC or CsgA preformed fibrils at 37 °C in the presence of 0 to 7.5 µm of Aβ monomers (*n* = 3). D) TEM images of FapC and CsgA fibrils incubated with or without Aβ (3.5 µm), FapC incubated with Aβ shows small amorphous species whereas short length fibrils were produced from CsgA' interaction with Aβ (scale bar: 100 nm). E) Dynamic light scattering (DLS) was performed to further characterized the change in fibrils size (*n* = 3). Both FapC or CsgA with Aβ showed significant size reduction (**, *p* < 0.005). F) In CD spectra, FapC or CsgA incubated with Aβ showed negative peaks at 222/212 nm and 222/208 nm respectively, whereas the fibrils incubated without Aβ showed minima at 214 nm. Thus, showing that Aβ induces a noticeable transition from β‐sheet to α‐helices. G) Secondary structures of FapC and CsgA alone and with Aβ. In FapC fibrils incubated with Aβ, β‐sheet content was reduced to 32% from 47% whereas for CsgA fibrils, β‐sheet content was decreased from 42% to 39%. A11 oligomers and anti‐Aβ immunostaining (H) and fluorescence quantification (I) of FapC + Aβ mixture showed positive staining for Aβ while negative for A11. Aβ oligomers formed under similar in vitro conditions were used as a positive control for A11 staining. J,K) FapC and CsgA binding affinity with Aβ was analyzed with ELISA and the dissociation constant (*K*_d_) was calculated. CsgA showed significantly higher (22.9 ± 4.5 µm) dissociation with Aβ in comparison to FapC 6.2 ± 1.9 µm (*, *p* < 0.05).

FapC and CsgA were fibrillated in vitro in deionized water at a concentration of 1 µg mL^−1^ at 37 °C for 2 weeks. The formation of fibrils was confirmed using transmission electron microscopy (TEM). We then investigated whether Aβ (Aβ1‐42) could dissociate microbial amyloid fibrils. FapC and CsgA fibrils (50 µm) were labelled with ThT dye and then incubated with 0 – 7.5 µm of Aβ monomers while ThT fluorescence was recorded at regular intervals for 45 h. With Aβ concentrations above 1.8 µm, a reduction in ThT fluorescence was observed in the first 15 h for both FapC and CsgA amyloids which subsequently plateaued after 15 h (**Figure** **1**A,B). Aβ concentrations of 0.45 and 0.9 µm induced negligible to an intermediate reduction in the ThT fluorescence. Aβ alone at equivalent concentrations exhibited no significant change in ThT fluorescence (Figure 1C), ruling out any ThT interference from Aβ. After performing the ThT assay, the break‐down products of FapC and CsgA amyloids were further analyzed using TEM (Figure 1D). Whereas original FapC and CsgA fibrils present with a mature amyloid morphology, incubation with Aβ resulted in complete break‐down of the FapC fibrils into amorphous debris (≈50 nm), and partial re‐modelling of CsgA fibrils into short‐truncated filaments of 100 – 200 nm length. The size reduction was confirmed with dynamic light scattering, which revealed a reduction in the hydrodynamic radius of FapC fibrils from ≈550 to 75 nm, and CsgA fibrils from ≈1500 to ≈210 nm (Figure 1E). We further analyzed the FapC and CsgA fragments, digested by Aβ, through SDS‐PAGE. The digested fragments of FapC and CsgA appeared as monomers of the proteins in SDS‐PAGE (Figure S1A,B, Supporting Information), however, the protein extracted from the bands appeared as a mixture of aggregated debris and monomers under TEM imaging (Figure S1C,D, Supporting Information). Circular dichroism (CD) spectroscopy revealed FapC and CsgA fibrils to have a characteristic negative peak for β‐sheets at 214 nm (Figure 1F). However, after incubation with Aβ, FapC + Aβ presented negative peaks at 222 and 212 nm, while CsgA + Aβ displayed distinct peaks at 222 and 208 nm – indicating a transition from β‐sheet to α‐helices. To further investigate whether this transition from β‐sheet to α‐helices is either due to the physical presence of Aβ peptide or the disintegration of FapC or CsgA fibrils, we analyzed the freshly mixed solution of Aβ and FapC or CsgA (Figure S2, Supporting Information). The freshly prepared mixture of microbial amyloids and Aβ presented a broad negative peak of β‐sheets at 214 nm, similar to the CD spectrum of FapC and CsgA fibrils alone, indicating that transition to α‐helices in FapC + Aβ or CsgA + Aβ samples are due to disintegration of FapC or CsgA fibrils into non‐fibrillar species over time. FapC fibrils incubated with Aβ (FapC + Aβ) lost the positive peak at 192 nm, further indicating the formation of random coils. When CD spectra were quantified for secondary structure content, β‐sheet contents for FapC fibrils were reduced from 47 to 32% and 42 to 39%, respectively for CsgA fibrils, after incubation with Aβ (Figure 1G). Random coil content for FapC fibrils were increased from 22 to 31%, indicating a significant dissociation or remodeling of FapC fibrils in the presence of Aβ. Interestingly, although the FapC broken fragments lost their amyloid morphology, they still retained some β‐sheet content. Both FapC and CsgA broken fragments presented a comparable increase in the helical content. To investigate the binding state of Aβ peptide with microbial amyloid fibrils, we performed A11 (oligomer specific) and Aβ‐peptide immunostaining on FapC + Aβ samples. FapC fibrils were mixed with Aβ and incubated for 4 h to allow binding between two species. Immunostaining revealed Aβ monomers adsorbed on FapC fibrils, instead of A11 positive Aβ oligomers (Aβo) (Figure 1H,I). To probe binding affinity to Aβ, we used ELISA to analyze the dissociation constant (K_d_) between Aβ and CsgA or FapC fibrils. Different concentrations of Aβ (ligand) were added to the polystyrene well where FapC or CsgA fibrils (20 µm) were immobilized. The binding of Aβ was quantified using anti‐Aβ42 and horseradish peroxidase linked antibodies and the binding curve is presented in Figure 1J. Dissociation constants (K_d_) of 6.2 ± 1.9 and 22.9 ± 4.5 µm for FapC and CsgA, respectively, were quantified through linear regression analysis (Figure 1K).

In contrast to Aβ, human islet polypeptide (IAPP) and α‐Synuclein (aSyn) did not show any disintegration of FapC amyloids at different tested concentrations (1.8 – 15 µm), for both IAPP and aSyn when measured against 50 µm of FapC amyloids. This was further confirmed by TEM and ThT measurements (Figure S3, Supporting Information). The ThT assay was performed for 72 h and TEM was performed at 1, 2‐, and 3‐week intervals to capture any remodeling event that may have occurred at a later stage. However, no remodeling effect was observed for FapC fibrils in the presence of IAPP or aSyn monomers.

### 2.2 Aβ can Dissolve the Biofilms Made through Live Culture of *P. aeruginosa* and *E. coli*

**Figure 2:**
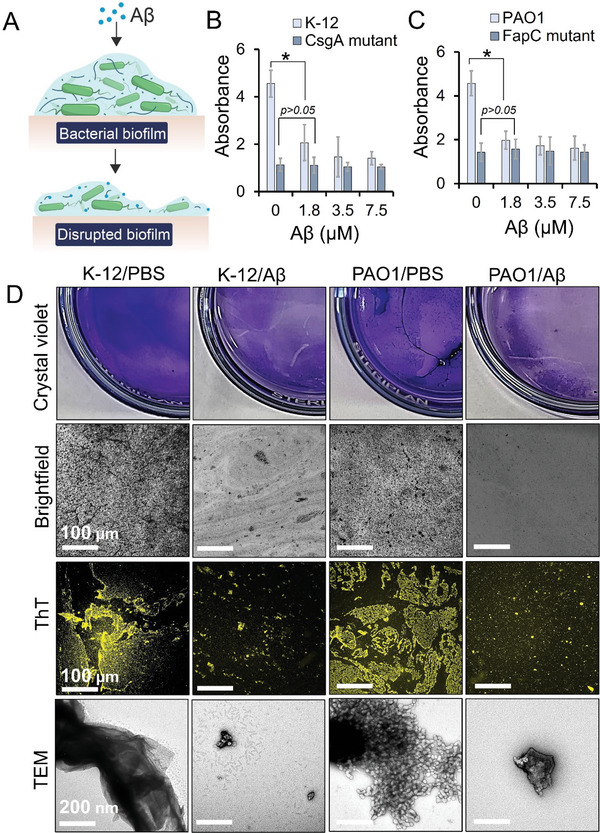
Aβ dissolved the biofilms made by the live culture of *P. aeruginosa* (PAO1) and *E. coli* (K‐12). A) Schematic illustration of Aβ dissolving microbial biofilms. B,C) K‐12, PAO1, CsgA mutant (MC4100), and FapC mutant (Pseudomonas UK4) cultures (optical density OD 0.3) were grown for 48 h in the wells of 96‐well plates with peg lid and biofilms were formed on the pegs. The biofilms were incubated with Aβ (0–7.5 µm) and stained with crystal violet dye (*n* = 3). Aβ at 3.5 µm significantly reduced biofilm synthesis (*, *p* < 0.05). D) PAO1 and K‐12 microbial cultures were grown overnight on small (100 mm) glass petri dishes. Biofilms formed at the glass surfaces were gently washed with PBS and treated with Aβ (3.5 µm) for 24 h (37 °C). After incubation with Aβ, biofilms were treated with 0.1% crystal violet stain for 15 min and images were taken by a stereomicroscope and brightfield channel (scale bar: 100 µm). Biofilms treated with Aβ were disintegrated in contrast to PBS control. ThT (100 µm, 15 min) labelled biofilms showed similar biofilm breakage into smaller fragments with Aβ (scale bar: 100 µm). Similarly, TEM was used to examine the fragmented morphology of disintegrated microbial biofilms treated with Aβ. K‐12 showed 400–500 nm and for PAO1 100–200 nm biofilm chunks were observed (scale bar: 200 nm).

To translate the results from FapC and CsgA amyloids into impact on actual biofilms made by *P. aeruginosa* (PAO1) and *E. coli* (K‐12), microbial cultures (optical density OD 0.3) were grown for 48 h in a 96‐well plate with Peg lid (Calgary biofilm device). YESCA media was used to facilitate the FapC and CsgA dependant biofilm formation and biofilms were formed on the pegs. The biofilm containing pegs were gently washed with PBS, incubated with 0–7.5 µm of Aβ for 12 h at 37 °C and biofilms were quantified via crystal violet assay. (**Figure** **2**A–C). The anti‐biofilm effect of Aβ was most evident at 3.5 and 7.5 µm concentrations in both K‐12 and PAO1. To confirm that disintegration of microbial biofilms was due to Aβ’ interaction with FapC or CsgA, we used FapC and CsgA mutant strains where no disintegration of biofilm was observed with Aβ (Figure 2B,C). These observations were extended to morphological imaging of the biofilms and we cultured PAO1 and K‐12 strains in glass petri dishes and the biofilms formed at the bottom of glass surface were gently washed with PBS twice to remove planktonic bacteria and subjected to a treatment with Aβ (3.5 µm) or PBS control for 24 h at 37 °C. The biofilms were stained with crystal violet dye and glass substrate of petri dishes were imaged under a stereomicroscope (Figure 2D). The biofilm treated with Aβ was disintegrated, as demonstrated by the lack of crystal violet staining. We exploited the underlying amyloid structure to stain the biofilms with ThT dye and image them in the green channel of a fluorescence microscope (510–560 nm),^[^ ^25^ ^]^ confirming the breakdown of microbial biofilms into smaller fragments. TEM imaging used to validate these findings also showed the fragmented morphology of the biofilm chunks (100 – 200 nm for both PAO1 and K12, respectively) following exposure to Aβ (Figure 2).

**Figure 3:**
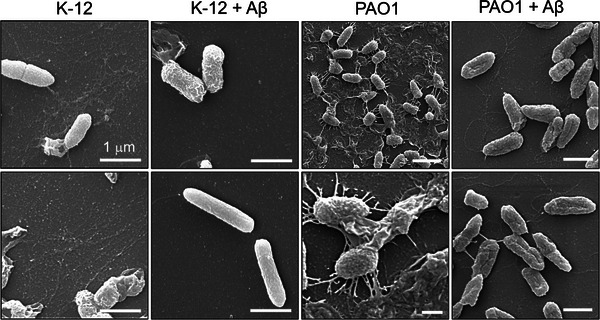
SEM imaging of PAO1 and K‐12 biofilms after treatment with Aβ. Microbial cultures treated with Aβ were further examined in SEM. K‐12 and PAO1 cultures treated with Aβ (3.5 µm) both revealed substantial reduction in fimbriae‐like biofilm structures in Aβ treated samples, as compared to PBS‐treated samples.

We further validated these results with scanning electron microscopy where biofilms were grown on a glass coverslip inside a glass petri dish and treated with Aβ (3.5 µm) under the same conditions. The coverslips were then subjected to SEM imaging which revealed the presence of fimbriae‐like fibrillar amyloids/biofilms for both untreated K‐12 and PAO1 microbial cells. Importantly, fimbriae‐like fibrillar amyloids/biofilms were not present in Aβ treated cultures (**Figure** **3**).

### 2.3 Aβ can Target and Co‐Localize with FapC and CsgA Amyloids In Vivo

**Figure 4:**
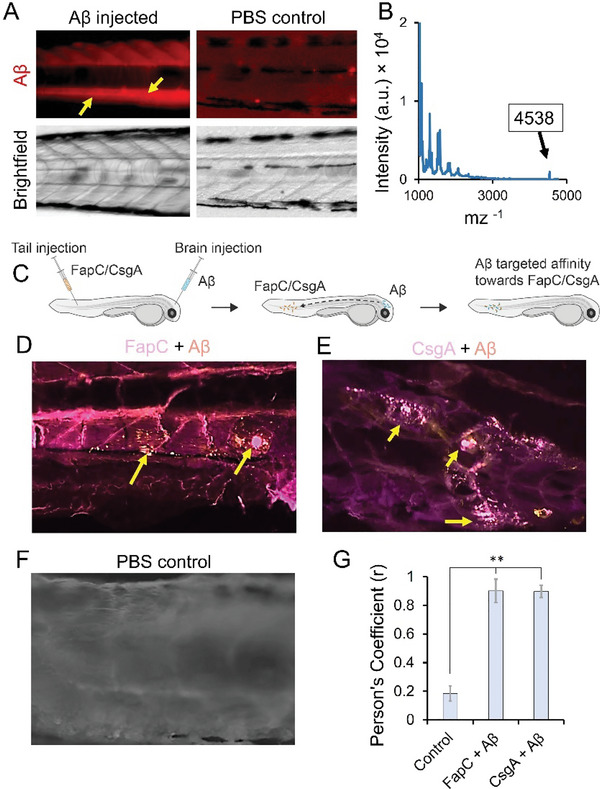
Aβ targets and co‐localises with FapC and CsgA amyloids in vivo. 5 days post‐fertilization (dfp) zebrafish larvae were selected and cerebrally microinjected (50 nL) with Alexa647 labelled 1 µm Aβ. A) Whole‐mount fluorescent imaging detected Aβ‐Alexa647 around main artery notochord and no fluoresce was observed in control larvae. B) In another group of 5 dfp zebrafish larvae, unlabelled Aβ peptide (50 nL, 1 µm) was cerebrally microinjected (*n* = 20). After 4 h trunk was separated from the head and homogenized. MALDI analysis from trunk regions detected Aβ peak at a molecular weight of 4538 mz^−1^. C) Microbial amyloids FapC or CsgA were labelled with Alexa488 and microinjected to the tail region of 5 dfp zebrafish larvae together with Aβ‐Alexa647 cerebrally microinjected. Larvae (lateral side) at 4 h post microinjection were fluorescently imaged, Aβ was found to be colocalized with D) FapC or E) CsgA, while no fluorescence was observed in control F). G) Pearson's Coefficient revealed significant colocalization of Aβ with FapC or CsgA (**, *p* < 0.005).

It has been proposed that Aβ can permeate across the blood‐brain barrier in both directions, i.e., blood‐to‐brain and brain‐to‐blood.^[^ ^26^, ^27^ ^]^ The blood‐to‐brain transport of Aβ has been demonstrated with circulating vascular Aβ^[^ ^28^ ^]^ while brain‐to‐blood transport is supported by the clearance mechanism of Aβ from the brain.^[^ ^29^ ^]^ Aβ has also been found diffusing into other organs such as the gut.^[^ ^13^, ^14^ ^]^ Therefore, we hypothesized that cerebral Aβ can target microbial biofilm amyloids in non‐neuronal tissues. Zebrafish develop a physiologically competent blood‐brain barrier system 3 days post‐fertilization (dfp).^[^ ^30^, ^31^ ^]^ Using 5 dfp zebrafish larvae, we first studied the vascular permeation of cerebrally injected Aβ. Aβ was labelled with Alexa647 and injected into the zebrafish larvae via cerebroventricular microinjection.^[^ ^4^, ^32^ ^]^ Fluorescence imaging of the larvae showed a visible circulation of Aβ‐Alexa647 in the main artery around the notochord at 4 h post‐injection. Notably, no fluorescence was observed in the circulation of the untreated control larvae (**Figure** **4**A). To further confirm transpermeation of cerebrally injected Aβ into the microvasculature of zebrafish larvae, an additional group of 5 dfp larvae was cerebrally microinjected with unlabelled Aβ peptide and Aβ identified by MALDI analysis of homogenized trunk tissues through a peak at 4538 mz ^−1^ (Figure 4B). To study the co‐localization of Aβ with microbial amyloids, Alexa 488 labelled FapC or CsgA amyloids were injected to the tail muscle of 5 dpf zebrafish larvae while Alexa 647 labelled Aβ was administered concurrently via cerebral microinjection (Figure 4C). After 4 h, Aβ was found to have travelled through the bloodstream and was visibly co‐localized with the injected FapC or CsgA fibrils (Figure 4D–F). Colocalization was further quantified via Pearson's Coefficient (Figure 4G). FapC and CsgA alone as controls are presented in Figure S4A (Supporting Information). In addition to the colocalization, a reduction in the fluorescence of FapC (Alexa 595 labelled) was also observed over a period of 24 h, in the larvae that was co‐injected with FapC to the tail muscle and Aβ to the cerebroventricular brain tissue (Figure S4B, Supporting Information).

### 2.4 Aβ Increases the Anti‐Microbial Susceptibility of *P. aeruginosa* and *E. coli*

**Figure 5:**
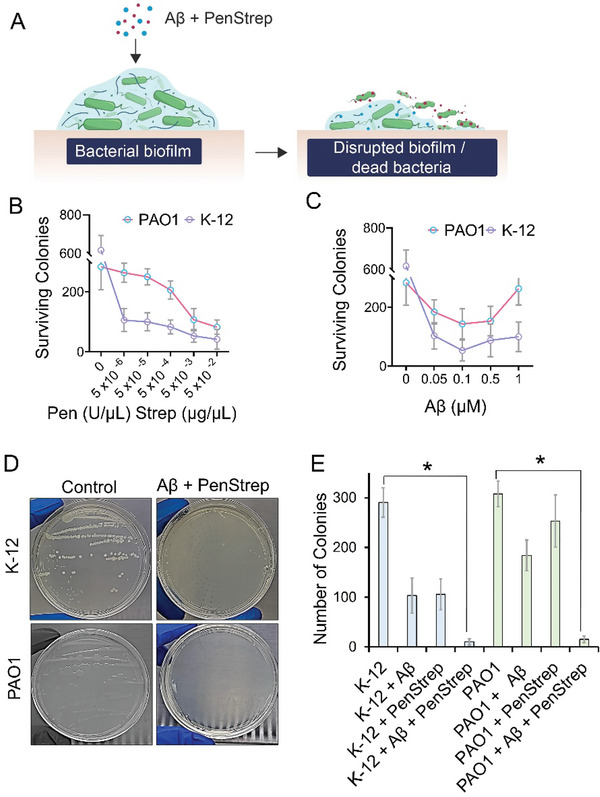
A) Aβ increases the anti‐microbial susceptibility of *P. aeruginosa* and *E. coli*. Microbial cultures of PAO1 and K‐12 were grown overnight (OD 0.3, at 37 °C) and subjected to series of dilutions of Aβ (0–1 µm) and Pen (0–5 × 10^−2^ U mL^−1^) Strep (0–5 × 10^−2^ µg mL^−1^) in LB media (*n* = 6). Biofilm swabs form pegs were streaked on LB agar plate and number of surviving colonies were counted. B) Concentration‐dependent antimicrobial effect with Pen, Strep effect was evident, C) whereas Aβ antimicrobial effect was diminished at higher concentrations. To examine the anti‐microbial susceptibility of PAO1 and K‐12, microbial cultures were treated overnight with selected concentrations of Aβ (0.05 µm) mixed with Pen (5 × 10^−6^ U mL^−1^) Strep (5 × 10^−6^ µg mL^−1^) in LB media. Biofilm swabs were streaked on LB agar plate (D) that presented a significant reduction in the number of surviving colonies (E) (*, *p* < 0.05).

As the previous results demonstrated that Aβ can remodel microbial amyloids, we further studied the impact of this remodeling/amyloid break‐down on the survival of microbial colonies, when exposed to a combination of Penicillin and Streptomycin (PenStrep) (**Figure** **5**A). Penicillin is a β‐lactam while Streptomycin is an aminoglycosides antibiotic and both are reported to face anti‐microbial resistance either due to poor penetration in biofilms or due to inactivation by β‐lactamase‐ an enzyme that is synthesized by bacteria and distributed to the biofilms.^[^ ^33^, ^34^ ^]^ A culture of *P. aeruginosa* or *E. coli* was grown in LB media in a 96‐well plate. The lid of the plate was fitted with pegs (Calgary Biofilm Device, CBD) that dipped into the LB media and microbial culture formed biofilms on those pegs using them as a substrate. The biofilms grown for 24 and 48 h showed a comparable formation of mature biofilms on the pegs (Figure S5, Supporting Information), however, literature reports 18–24 h time point for 96‐well CBD method,^[^ ^35^, ^36^, ^37^ ^]^ therefore, biofilm grown for 24 h on the pegs were used for this experiment. Subsequently, the biofilms made on the peg were challenged by dipping in another 96‐well plate with LB media, containing a series of dilutions of Aβ or PenStrep. After challenging the biofilms with Aβ or PenStrep, swabs of the biofilms from the pegs were streaked on LB agar plates to grow any surviving colonies (Figures S6 and S7, Supporting Information). A concentration‐dependent anti‐microbial effect for PenStrep was observed (Figure 5B) while, in the case of Aβ, the antimicrobial effect was reduced at higher concentrations (Figure 5C). A concentration of 5 × 10^−6^ U mL^−1^ (Pen) and 5 × 10^−6^ µg mL^−1^ (Strep) was selected as it was a minimum concentration that demonstrated a slight antimicrobial effect. The PenStrep was then mixed with 0.05 µm of Aβ to make another challenge solution in LB media. Biofilms of *P. aeruginosa* and *E. coli* were grown on the pegs of a 96‐well plate lid and then immersed in the challenge solution of PenStrep + Aβ for 24 h. Swabs from the pegs were streaked on agar plates (Figure 5D). No surviving microbial colonies were detected (Figure 5E), indicating that Aβ enhanced the antimicrobial sensitivity of microbial colonies. The results in Figure 5B,C and Figure 2 represent antimicrobial and antibiofilm activities independently from each other as in both cases, preformed mature biofilm was subjected to Aβ treatment. Therefore, any microbial cells being targeted by Aβ during biofilm quantification assay (Figure 2) should not interfere in the results from the pre‐formed biofilm. Another observation is that a low concentration of Aβ can disintegrate FapC or CsgA fibrils (1.8 µm Aβ, Figure 1A,B) and increase the susceptibility of microbial biofilms (50 nm of Aβ, Figure 5E) to antibiotics. These observations can be attributed to repeated interaction or the presence of multiple binding motifs on Aβ that enable their interaction with FapC or CsgA. Also, in the case of FapC and CsgA, the fibrils were disintegrated into shorter fibrils or smaller aggregates with intact β‐sheets (Figure 1F,G), which also support the feasibility of a smaller concentration of Aβ to disintegrate FapC and CsgA fibrils or microbial biofilms.

### 2.5 The Cellular Interaction and Cross‐Seeding Properties of Remodeled Fragments of FapC and CsgA Amyloids

**Figure 6:**
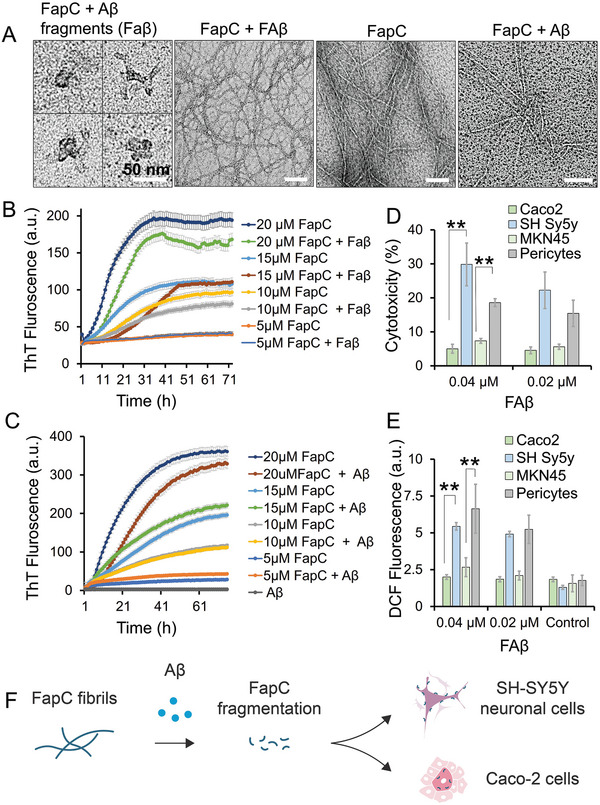
The cross‐seeding properties of remodeled fragments of FapC amyloids against FapC monomers. A) After Aβ treatment, the remodeled FapC fragments (Faβ) were collected purified, concentrated and imaged under TEM (scale bar: 50 nm). FapC fibrils grown in the presence or absence of Faβ did not show any difference, presenting no impact of Faβ on FapC fibrils formation. However, Faβ equivalent Aβ concentrations have a slight remodeling effect on the final FapC fibrils. B) Slight suppression in the ThT kinetics of FapC was observed in the presence of Faβ, however, that was also observed C) in the presence of Aβ (0.19 µm equivalent to 20 µm Faβ). D) Faβ (0.02 – 0.04 µm) was incubated with Caco‐2 and SH‐SY5Y cell lines and cytotoxicity and E) ROS was measured. Faβ at exhibited a significant level of cytotoxicity and ROS to SH‐SY5Y cells as compared to Caco‐2 cells (**, *p* < 0.005). F) Schematic illustration of differential toxicity of Faβ toward SH‐SY5Y and Caco‐2 cells.

Our in vitro results (Figure 1) indicate that, in contrast to CsgA amyloids, Aβ can efficiently disintegrate/remodel FapC amyloids, breaking them into non‐uniform fragments (Faβ). As Aβ can co‐localize with microbial cells in the gut^[^ ^13^ ^]^ and our results suggest that Aβ can target microbial amyloids in the tissues outside the brain and remodel them in vitro, it was reasonable to study the effect of this remodeled by‐product on mammalian cells. Therefore, next we studied the cellular interaction of Faβ with neuronal (SH‐SY5Y) and intestinal (Caco‐2) cells. Here, Faβ appeared to present an irregular morphology (**Figure** **6**A). However, some of the β‐sheet architecture was retained in vitro (Figure 1F,G). This finding provided the rationale for us to study whether Faβ can self‐seed and initiate the growth of new FapC amyloids. Faβ were collected following incubation of FapC with Aβ and purified through centrifugal filters (0.2 µm pore size). We found that Faβ, when incubated with different concentrations of FapC monomers, had no impact on the morphology of the resulting FapC fibrils when compared with the FapC fibril control (Figure 6A). We anticipated that some Aβ‐Faβ attachment would skew the results. We, therefore, chose to incubate FapC monomers with Faβ equivalent concentrations of Aβ. Here, we observed no impact on the morphology of FapC fibrils, ruling out this possibility.

**Figure 7:**
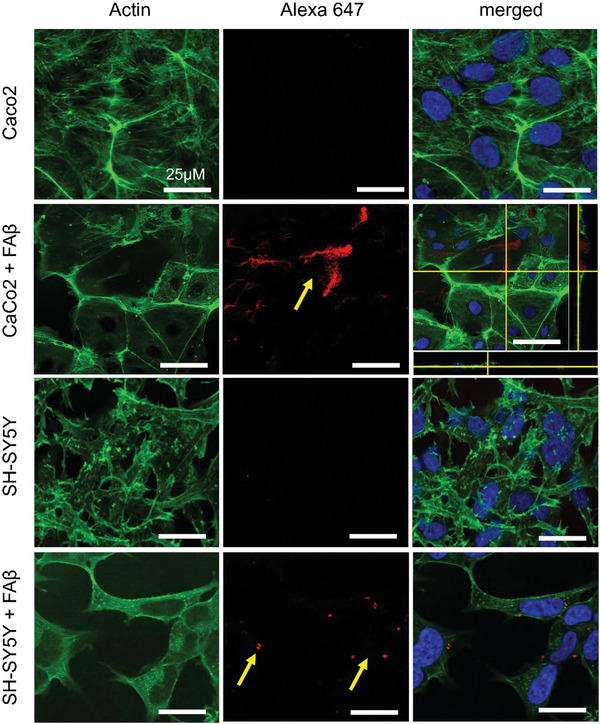
Internalization of Faβ fragment in CaCo‐2 and SHSY‐5Y cells. Faβ were labelled with Alexa647 and subjected to Caco‐2 or SH‐SY5Y cells. After 4 h, cells were imaged under the confocal microscope. Z‐stack images showed Faβ internalization into CaCo‐2 cells, however, in SH‐SY5Y cells, Faβ were found binding to the membrane.

Although the ThT assay presented a slight suppression of FapC fibrillization kinetics in the presence of Faβ (Figure 6B), similar suppression in FapC fibrillization was observed in the presence of 0.19 µm Aβ (equivalent to 20 µm of Faβ), however, insignificant increase in the ThT curve of FapC was observed with Aβ concentration lower than 0.19 µm (Figure 6C). We further explored Aβ‐remodeled FapC (Faβ) and CsgA (Caβ) fragments to study whether they can self‐seed the growth of respective FapC or CsgA monomers (Figure S4, Supporting Information). No seeding effect of Faβ or Caβ was observed against FapC or CsgA in different concentration ratios. Sonicated FapC or CsgA seeds and non‐sonicated full‐length FapC or CsgA fibrils were used as control where only sonicated FapC, self‐seeded the growth of FapC monomers and a significant increase in ThT kinetics was observed (Figure S8, Supporting Information). Cytotoxicity experiment showed toxicity and ROS damage when Faβ (0.04 and 0.02 µm) was exposed (24 hr) to SH‐SY5Y cells and immortalized human pericytes, but no toxicity or ROS was observed with Caco‐2 and gastric MKN45 cells (Figure 6D–F). To further explore the basis for these differences, we labelled Faβ with Alexa647 and administered it to Caco‐2 or SH‐SY5Y cells for 4 h. After subsequent fixation, we imaged the cells using fluorescence confocal microscopy. Faβ was observed in the cytoplasm of Caco‐2 cells, whereas for SH‐SY5Y cells, it appeared as aggregates mainly localized at the cell membrane. This differential interaction can help explain the selective toxicity toward SH‐SY5Y cells and highlights a possible toxic disruption of normal membrane function in neuronal cells (**Figure** **7**).

### 2.6 Aβ‐Digested FapC (Faβ) and CsgA (Caβ) Amyloids have Reduced Cellular Adhesion

**Figure 8:**
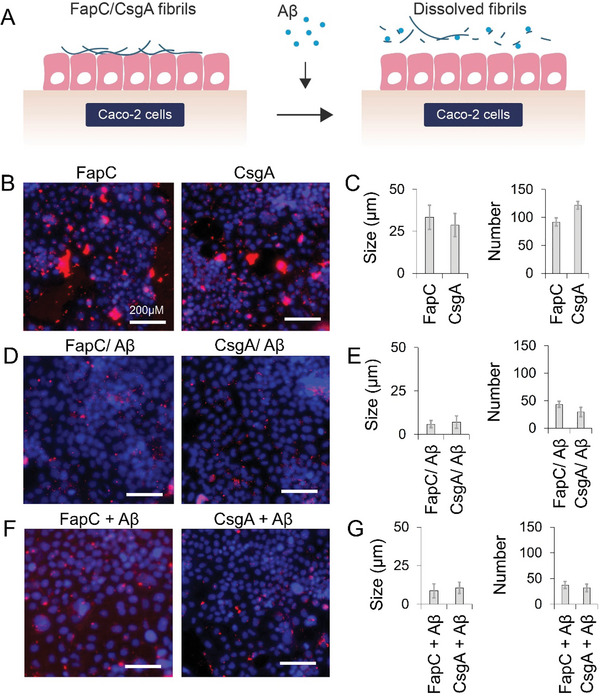
A) Aβ‐remodeled FapC and CsgA amyloids have lost cellular adhesion. B) Ten micrometer FapC or CsgA amyloids (Alexa647 labelled) were incubated with CaCo‐2 cells and imaged attached to the cells after 24 h. C) The size and numbers of FapC or CsgA amyloids clusters, attached to the cell membrane, were quantified. The cells were incubated with Alexa647 labelled FapC or CsgA amyloids for 24 h and then with Aβ for additional 24 h. The results (D) demonstrate that Aβ was able to remodel FapC or CsgA amyloids on the surface of cells and the size and number of the amyloid clusters were reduced E). F,G) Similar results were observed with FapC or CsgA amyloids, pre‐remodeled by Aβ were incubated with the cells, i.e., FapC or CsgA amyloids lost their cell adhesion ability after remodeling by Aβ.

To assess the cellular adhesion property of FapC or CsgA amyloids, before and after treatment with Aβ (**Figure** **8**A), we incubated Alexa647 labelled FapC or CsgA amyloids with Caco‐2 intestinal cells. Cells were fixed in paraformaldehyde and imaged using immunofluorescent confocal microscopy at 24 h post incubation. FapC or CsgA amyloid aggregates ≈30 µm in size were observed attaching to the cellular membrane (Figure 8B,C). When we incubated Alexa647 labelled FapC and CsgA amyloids with Caco‐2 cells for 24 h and then added 1 µm Aβ for an additional 24 h, smaller‐sized fragments of 5–8 µm of microbial‐amyloids were observed on the Caco‐2 cells and the number of fluorescence spots was significantly reduced. This indicates that Aβ was able to recapitulate the *in vitro* remodeling of FapC and CsgA amyloids on the surface of the cell membrane and reduced their cellular adhesion (Figure 8D,E). Similar results showing reduced cell adhesion were observed with Faβ and Caβ that were pre‐incubated with Aβ before introducing them to the cells (Figure 8F,G). This data supports the notion that Aβ can interact and digest/remodel microbial amyloids that are already attached to the cell membrane and that broken‐down fragments of FapC and CsgA amyloids lose their cell adhesion ability. The cell culture media of FapC, CsgA, FapC/Aβ and CsgA/Aβ samples were imaged to find the fate of fluorescent labelled FapC or CsgA fibrils, after digestion with Aβ. The higher fluorescence in FapC/Aβ and CsgA/Aβ, in contrast to FapC or CsgA controls indicated the disintegrated fibrils (fluorescent labelled) were released back into the media (Figure S9, Supporting Information).

## 3 Discussion

In the quest to unravel the physiological role of Aβ, the anti‐microbial hypothesis of Aβ is emerging as an interesting proposal. Fibrillization of Aβ into oligomers and amyloid fibrils is associated with the neurodegeneration of Alzheimer's disease and is also reported to have implications in other protein‐aggregation diseases through similar or dis‐similar cross‐seeding.^[^ ^38^, ^39^, ^40^, ^41^ ^]^ Research indicates that Aβ oligomers can bind to the carbohydrates of the microbial cell wall through sequence‐specific motifs and induce antimicrobial activity,^[^ ^13^ ^]^ however, Aβ oligomers are also considered as the toxic species that drive Alzheimer's neurodegeneration. Therefore, the physiological role of Aβ monomeric peptide remains unclear. The association of Aβ peptide with the gut tissues^[^ ^14^, ^15^ ^]^ can unravel another physiological aspect of Aβ, i.e., its role in mediating the communication along the gut‐brain axis. Opportunistic pathogens such as *E. coli* and *P. aeruginosa* use specific amyloid proteins CsgA and FapC to make their biofilms. Both bacteria have demonstrated independent infectious and pathogenic roles inside and outside the intestine.^[^ ^42^, ^43^ ^]^

### 3.1 Aβ can Biophysically Disintegrate the Microbial Amyloids In Vitro and in Microbial Biofilms

The disintegrative role of Aβ was more intense toward FapC, which was broken down to smaller 50 nm debris, while CsgA amyloids merely formed smaller filaments of 100–200 nm. One possible explanation for this difference could be the higher positive charge on FapC fibrils (47 mV) when compared to 37 mV on CsgA fibrils. Aβ monomers with a zeta potential of −1.5 mV might show greater binding affinity toward FapC fibrils on purely electrostatic grounds (Figure S10, Supporting Information). This became evident in the ELISA assay, where Aβ demonstrated a higher binding affinity for FapC (dissociation constant; *K*_d_ 6.2 µm) than CsgA fibrils (*K*_d_ 22.9).

Fragmented amyloids of CsgA and FapC were able to retain their internal β‐sheet structure, as identified in CD. This indicated that Aβ was remodeling or disintegrating microbial amyloids, rather than performing a complete digestion role. In a live microbial culture, amyloid fibrils are coated with DNA, lipids and carbohydrates that may shield the underlying amyloid scaffold from interacting with Aβ. Therefore, we attempted to fully recapitulate the interaction between Aβ and FapC or CsgA amyloids using *P. aeruginosa* or *E. coli biofilms*. Aβ was able to dissolve the preformed microbial biofilms as discovered using fluorescence and electron microscopy, and biofilm assays. In addition, Aβ was able to break down FapC and CsgA amyloids that were pre‐coated with a mixture of bovine serum albumin, cholesterol, and glucose (Figure S11, Supporting Information). The FapC mutant (*Pseudomonas UK4)* and CsgA mutant (MC4100) strains didn't show biofilm disruption upon exposure to Aβ, indicating the biofilm disruption is dependent on Aβ’ interaction with microbial amyloids. The control *Pseudomonas UK4* (FapC expressing) showed similar disintegration of biofilms, upon exposure to Aβ, as observed with *P. aeruginosa* PAO1 (Figure S12, Supporting Information).

Although Aβ is produced in the cerebral tissues, it can also be found co‐localized with the microbes in the gut.^[^ ^13^ ^]^ Such literature indirectly indicates the probability that Aβ can sustain proteolytic cleavage by enzymatic degradation in the gut. To probe this feasibility, we have exposed Aβ peptide to trypsin, a proteolytic enzyme from the gut. After 6 h of incubation, Aβ was detectable in the mixture (Figure S13, Supporting Information). Both in Alzheimer's patients and transgenic mouse models of the disease, Aβ aggregates are found locally in various enteric systems.^[^ ^12^, ^13^ ^]^ Also, Aβ injected to the intestinal viscera of the mice can induce Aβ aggregation in the brain.^[^ ^15^ ^]^ Taken together, it can be hypothesized that Aβ can permeate across the blood‐brain barrier from either side. Indeed, this was evidenced in our experiments, where Aβ injected to the brain of the wild‐type zebrafish larvae, was able to permeate into the blood vasculature and co‐localize with the FapC or CsgA amyloids injected at the site of the tail muscle.

### 3.2 Microbial Amyloids Remodeled by Aβ Lose the Cell‐Adhesion Ability and Increase Anti‐Microbial Susceptibility

In microbial culture, Aβ at a dose of 50 nm was able to increase the susceptibility of *E. coli* and *P. aeruginosa* toward Penicillin and Streptomycin antibiotics. The concentration of Aβ beyond 100 nm reduced its anti‐biofilm activity, which can be explained based on the critical aggregation and fibrillization concentrations of Aβ being higher than 90 and 200 nm, respectively.^[^ ^44^, ^45^ ^]^ Therefore, an enhanced Aβ‐Aβ interaction (i.e., for self‐assembly or aggregation) at concentrations higher than critical aggregation concentration can competitively overcome Aβ‐biofilm interaction.^[^ ^46^, ^47^ ^]^ The broken or sonicated fragments of amyloid fibrils can cross‐seed the growth of further amyloids via cross‐β‐sheets interactions^[^ ^32^, ^48^, ^49^ ^]^ and disintegrated fragments of FapC (Faβ) and CsgA (Caβ), achieved through Aβ treatment, retained their internal β‐sheet structure. Therefore, we assessed whether Faβ or Caβ can self‐seed the growth of new FapC amyloids. We observed that Faβ or Caβ were not able to self‐seed FapC or CsgA monomers into amyloids. However, Faβ was able to interact with the membrane of the neurons and induce cytotoxicity. In contrast, and as expected, intestinal Caco‐2 were able to internalize Faβ without a cytotoxic occurrence. Caco‐2 are intestinal epithelial cells that have previously reported to uptake prion‐like protein fragments, potentially through epical protein delivery mechanism or transferrin uptake receptors.^[^ ^50^ ^]^ Enzyme‐assisted partially‐digested prion fragments were phagocytosed and transported across Caco‐2 cells monolayer.^[^ ^50^ ^]^ Such phagocytosis by Caco‐2 cells has also been demonstrated with *E. coli* virulence protein EspF.^[^ ^51^ ^]^ Finally, the interaction between Aβ and FapC or CsgA amyloids was recapitulated on the surface of intestinal cells where treatment with Aβ disintegrated FapC or CsgA amyloids that were already attached to the cell membrane. Also, the CsgA or FapC amyloids, pre‐disintegrated with Aβ, lost their ability to attach to the cell membrane. This suggests that the full length of mature microbial amyloids is necessary for their cellular adhesion. Cellular adhesion is one of the core functions of microbial biofilms, and this study demonstrated that Aβ can reduce cellular adhesion of microbial amyloids by disintegrating them into smaller fragments.

## 4 Conclusion

In summary, here we report for the first time a specific anti‐biofilm role for Aβ monomeric peptide, where it can remodel and disintegrate FapC and CsgA amyloids of *P. aeruginosa* and *E. coli¸* both in solution and when microbial amyloids were attached to the intestinal cells membrane. While Aβ has been studied permeating to and from the brain and interacting with the pathogens in the gut, this research also explains the role of Aβ in communications along the gut‐brain axis. Opportunistic pathogens such as *P. aeruginosa* and *E. coli* and their biofilms can participate in extra‐intestinal pathologies. Aβ could play a role as a native anti‐biofilm agent in the gut and/or the site of infection. The broken‐down fragments of FapC amyloids obtained by Aβ treatment, proved neurotoxic to SH‐SY5Y cells, while the uptake by intestinal Caco‐2 cells suggests a propagative pathogenic role for infectious biofilms stemming from the gut. As more alternative anti‐amyloid approaches evolve to target Alzheimer's disease and dementia, proper understanding of the physiological role of Aβ monomers will be crucial in helping to shape those therapeutic approaches. Notably, this study also demonstrates the anti‐biofilm role of Aβ at both local cerebral or distal sites in the human host such as gut or gut‐associated tissues, something that can also assist the future development of anti‐microbial therapies.

## 5 Experimental Section

### Animal Husbandry and Ethics Statement

The wild‐type zebrafish (AB, *Danio rerio*) was bred in a fish circulatory system at the University of Queensland Aquatic Facility at 28 ± 0.5 °C under a 14:10 h light and dark cycle. The zebrafish larvae were maintained in Holtfreter's buffer and 5 days post fertilization (dpf) larvae were used for microinjection. The microinjections were performed after anesthesia with 0.4% tricaine. Euthanasia was performed with ice‐chilled water and heads were separated from the trunk with a surgical knife and washed thrice in phosphate‐buffered saline (PBS, pH 7.4). All in vivo experiments with larvae were performed according to the Occupational Health & Safety (OHS) guidelines of the University of Queensland. No ethics approval was required for using zebrafish larvae of the age of ≤5 dpf.

### Synthesis of FapC and CsgA fibrils

FapC and CsgA monomers, prepared recombinantly in *E. coli* as described,^[^ ^52^, ^53^ ^]^ were treated with hexafluoro‐2‐propanol (HFIP) and formic acid (50:50, 3 h) to dissolve preformed any oligomeric aggregates. Monomers were aliquoted and freeze‐dried. FapC or CsgA monomers (1 mg mL^−1^) were dissolved in deionized water and incubated at 37 °C for 2 weeks to produce mature fibrils. The formation of mature fibrils was confirmed with TEM. These fibrils were stored at 4 °C and used for further experimentation after bringing them to room temperature. The purity of Aβ peptide and FapC and CsgA proteins were confirmed through mass spectrometry (Figure S14A, Supporting Information) and SDS‐PAGE (Figure S14B, Supporting Information).

### Thioflavin T (ThT) assay

Aβ (Anaspec) was dissolved in HFIP and incubated at room temperature for 3 h to dissolve any aggregates to monomeric form. Aβ was aliquoted, freeze‐dried and stored for further experimentation. For ThT kinetics assays, different concentrations of Aβ (0 – 7.5 µm) were dissolved in 2 µL of DMSO, diluted with water and mixed with the aqueous solution of FapC or CsgA fibrils (50 µm). FapC and CsgA fibrils were pre‐incubated with 50 µm of Thioflavin T dye for 3 h. Twenty microliter of the solution containing Aβ, FapC or CsgA fibrils and ThT dye was added to a 384 well micro‐plate and incubated inside a microplate reader (37 °C), set at recording the ThT fluorescence (excitation 440 nm and emission 485 nm) at specified time intervals (10 min). The instrument was set to agitate the plate for 5 s before every reading. The ThT fluorescence was recorded for 45 h.

### Transmission Electron Microscopy (TEM)

FapC and CsgA fibrils (50 µm) were incubated with Aβ (3.5 µm) for 48 h and 5 µL of this solution was placed on a glow‐discharged carbon‐coated copper grid for 1 min. The samples were blotted with filter paper and grids were blotted twice with deionized water and then a drop of 1% uranyl acetate was placed on the grids for 30 s for negative staining. FapC or CsgA fibrils incubated without Aβ were used as controls. FapC or CsgA fibrils (50 µm) were incubated with a mixture of glucose (5 µm), cholesterol (5 µm), and bovine serum albumin (1 µm) at 37 °C overnight and then incubated with Aβ (7.5 µm) under same conditions. TEM was performed after 48 h of incubation. TEM grids were imaged with a Hitachi HT7700 TEM operating at 120 kV.

### Dynamic Light Scattering (DLS)

DLS was used to measure the change in size and zeta potential for FapC and CsgA fibrils, after incubation with Aβ. Full‐length FapC and CsgA fibrils were incubated with or without Aβ monomers under the same conditions as for TEM. Quartz cells with low volume capacity (100 µL) were used and measurements were performed using a Malvern Zetasizer Nano Series running DTS software and using a 4 mW He−Ne laser operating at a wavelength of 633 nm and equipped with an avalanche photodiode (APD) detector. The scattered light was detected at an angle of 173°.

### Sodium Dodecyl‐Sulfate Polyacrylamide Gel (SDS‐PAGE) Electrophoresis and Protein Extraction

FapC and CsgA fibrils (30 µg) were incubated with Aβ (5 µg) for 48 h at 37 °C. Following the incubation samples were mixed with SDS loading dye (with added dithiothreitol) for 5 min at 95 °C and separated using Bio‐Rad 4–20% (w/v) SDS‐PAGE PROTEAN® TGX™ Precast Protein Gel. FapC or CsgA fibrils incubated without Aβ were used as controls and a protein ladder (Bio‐Rad) was run alongside the samples. Gel electrophorese was performed at 60 v for the first half, then increased to 100 v until samples had reached the end. The gel was removed from the electrophoresis tank and stained with Coomassie stain (Bio‐Rad). Protein bands were visualized, and images were captured using Bio‐Rad ChemiDoc MP Imaging System. Protein bands were then carefully excised from the gel using a sharp blade and stored in low‐protein binding microcentrifuge tubes. Isolated protein bands were crushed into small pieces and 250 µL of elution buffer (50 mm Tris‐HCl, 150 mm NaCl, 0.1 mm EDTA, pH 7.5) was added to the tubes for overnight incubation at 30 °C with 500 rpm in Eppendorf ThermoMixer. Following the incubation, samples were centrifuged at 10,000 g for 10 min and supernatants were collected and stored at 4 °C until visualized under TEM.

### Immunostaining

FapC fibrils (40 mm) were incubated with Aβ (7.5 mm) for 3 h at room temperature. Following the incubation 30 mL of samples were placed on poly‐L‐lysine coated glass slide. After 30 min, glass slide was gently washed with MiliQ water. The slides were blocked with 1% BSA that was gently poured off the slides after 10 min. Glass slides were labelled with primary antibodies (rabbit anti‐amyloid‐β42, 1/300 dilution: or rabbit anti‐amyloid‐β42 Oligomer A11, 1/300 dilution). After 3 h of incubation, glass slides were washed with MiliQ water and incubated with secondary goat Anti‐rabbit IgG antibody (Dylight 594, 1/1000 dilution) for 30 min in the dark. Slides were washed with MiliQ water, mounted with a cover slip and mounting medium and immediately imaged under the red fluorescence channel of an Olympus BX61 fluorescent microscope. Images were quantified for fluorescence intensity using Fiji ImageJ software.

### Circular Dichroism (CD) Spectroscopy

FapC or CsgA fibrils (50 µm) were incubated with or without Aβ (3.5 µm) for 48 h under the same conditions as for TEM and CD spectra was recorded with a Jasco CD spectrometer from 190 to 240 nm operating at 25 °C and 1 nm step size. The percentage secondary structure was calculated by the CD data via Dichroweb analysis with parameters of Contin/reference set 4. For CD spectroscopy, Aβ was dissolved in 5 µL of 0.1% NH4OH buffer instead of DMSO and then made up with deionized water for the rest of the experiment. For the CD spectra of freshly mixed FapC or CsgA fibrils and Aβ, similar concentrations of the fibrils and Aβ peptide were mixed and incubated for 30 min at room temperature before CD analysis.

### Enzyme‐Linked Immunosorbent Solid Phase Assays (ELISA)

EELISA was performed to measure the binding affinity between bacterial amyloids (FapC and CsgA) with Aβ using a previously described protocol.^[^ ^54^ ^]^ Briefly, FapC or CsgA fibrils (20 µm) were coated overnight onto the poly‐L‐lysine coated polystyrene microtiter 96‐well plate. The coated FapC or CsgA fibrils were blocked with 1% BSA. Aβ was dissolved in 0.1% DMSO and different concentrations (0 – 20 µm) were prepared in MiliQ water, added to the wells, and incubated for 6 h at 4 °C. After washing thrice with water, Aβ bound to the plate was detected with a rabbit anti‐amyloid‐β42 antibody and horseradish peroxidase (HRP) assay. After washing with water, the plates were incubated for 1 h with primary rabbit anti‐amyloid‐β42 (1/1000 dilution) antibody. Then anti‐rabbit horseradish peroxidase‐linked antibody kit (Amersham Life Science) was used to quantify Aβ by measuring the luminescence at 428 nm with a microplate reader. The data were analyzed by a nonlinear regression fit algorithm in Prism9 to calculate the kD values.

### Bacterial Biofilm Assay

*P. aeruginosa* strain PAO1, *E. coli* K‐12, *Pseudomonas UK4 (*FapC mutant or FapC expressing) and CsgA mutant (MC4100) culture were streaked on Luria‐Bertani (LB) agar and incubated overnight at 37 °C. A single colony was picked from the agar plate, inoculated in LB or YESCA liquid media and grown overnight at 37 °C. Fresh cultures of PAO1 and K12 were diluted in respective media for an optical density (OD 600) of 0.3 and incubated for 48 h in a 96 well microplate (with pegs, Calgary Biofilm Device) or glass petri dish (100 mm diameter) under static conditions. After incubation, the planktonic bacteria were pipetted out and pegs or petri dish was washed with PBS (pH 7.4). The biofilm formed on pegs or layer of biofilms formed at the bottom of glass petri dish was stained with 0.1% crystal violet. After 20 min crystal violet dye was removed and the stained biofilm was washed three times with PBS (pH 7.4). For the crystal violet absorbance assay, 30% acetic acid was added to dissolve biofilm rings and absorbance (600 nm) was read using a multimode plate reader. Whereas glass petri dishes with and without crystal violet staining were subjected to optical imaging. For ThT staining of microbial biofilms, the biofilms were grown on the bottom of the glass petri dish, stained with 100 µm for 15 min, washed thrice with PBS and imaged under the green channel of a fluorescence microscope. To study the impact of Aβ on the preformed biofilms, once the biofilms were grown on the bottom of glass petri dishes, biofilms were washed with PBS thrice and then incubated with Aβ peptide (7.5 µm, dissolved in 2 mL PBS) and incubated for 12 h at 37 °C without shaking. After incubation, the petri dishes were gently washed with PBS thrice and subjected to crystal violet or ThT staining and imaging. PBS alone was used as a control. To study the effect of different concentrations (0–7.5 µm) of Aβ of biofilm formation, varying concentrations of Aβ were incubated with preformed biofilms in a 96 well microplate for 12 h, stained with crystal violet dye and biofilms were quantified as described above.

### Field Emission Scanning Electron Microscopy (FE‐SEM)

Bacterial biofilms were grown, as described above, in LB medium on a glass coverslip in glass petri dishes. After 48 h incubation, only planktonic bacteria were removed and Aβ (2 mL, 3.5 µm) was added to the petri dishes and incubated for additional 12 h. After incubation, samples were fixed overnight with 4% paraformaldehyde (PFA) and 2% glutaraldehyde in a 50:50 ratio. The next day samples were washed with fresh PBS and overnight air dried in Hexamethyldisilazane (HMDS) solution. The samples were mounted on a metal stub using a sticky carbon tape which increases conductivity and subjected to a 10 nm thick coating with platinum. Samples were imaged with JEOL JSM‐7001F 8KV FE‐SEM.

### Microinjections to Zebrafish Larvae

To study the permeation of Aβ from brain to the microvasculature, Aβ was labelled with Alexa647 (4:1 molar ratio) via 3 h incubation and washed with centrifugal filters (3 kDa MWCO) in the dark. Aβ (50 nL of 1 µm, labelled) was injected to the cerebroventricular space of 4 dpf larvae. The larvae were anesthetized in 0.4% tricaine before microinjection and then revived by placing them back into the Holtfreter's buffer. The microinjection was performed with a calibrated microneedle and pneumatic microinjection system (PV830 WPI), under a stereomicroscope. After 4 h post‐injection, the zebrafish larvae were anesthetized and placed in a droplet of low melting agarose (1.5% in Holtfreter's buffer) and subjected to whole‐mount imaging under an Olympus BX61 fluorescence microscope. In another set of experiments, FapC and CsgA fibrils (10 nL, 0.5 µm) were microinjected to the tail muscle of the zebrafish larvae, together with microinjection of Aβ to the brain. FapC and CsgA fibrils were pre‐labelled with Alexa488 (4:1 molar ratio) before microinjection. Four hours after injection with FapC or CsgA fibrils and Aβ, larvae were subjected to whole‐mount imaging as described above. The Person's colocalization coefficient was calculated with Fiji ImageJ software. FapC and CsgA alone as controls were labelled with Alexa 595 and microinjected to the zebrafish larvae using the same protocol. To study the reduction in the fluorescence intensity of labelled FapC fibrils from the tail muscle, the larvae were placed back into the buffer for 24 h in the dark and imaged the next day under a fluorescence microscope.

### Matrix‐Assisted Laser Desorption Ionization‐Time of Flight (MALDI TOF MS) Mass Spectrometry

The zebrafish larvae that were injected with Aβ (50 nL, 1 µm) were euthanized 4 h post‐injection. Trunks were separated from the head with a surgical blade and washed thrice in PBS. The trunks were placed into 100 µL of PBS and homogenized in a tissue homogenizer at 70 Hz for 2 min. This homogenate (1 µL) was mixed with a matrix (saturated solution of sinapinic acid (SA) in ethanol 2 µL) and 1 µL of this mixture was dried on a ground steel MALDI plate. The dried layer was analyzed by a Bruker ultraflextreme MALDI‐TOF/TOF in the linear positive mode where 8000 shots were performed across the sample spot. The acquired spectra were processed by baseline subtraction and peak picking using Flexanalysis software 3.4. For the trypsin stability study, the Aβ and trypsin (1% w/w) was incubated at 37 °C for 6 h and then subjected to MALDI analysis. The purity of Aβ peptide, supplied by Anaspec USA, was also studied through MALDI analysis using the same protocol.

### Antimicrobial Sensitivity Assay

All solutions used for anti‐microbial assays were sterilized by autoclaving or filtering except Aβ solutions that were prepared in DMSO/deionized water. Microbial culture of K12 and PAO1 were added to 96‐well plates at OD 0.3 and incubated overnight at the static condition at 37 °C with Minimum Biofilm Eradication Concentration (MBEC) peg lids (Innovotech, Edmonton, Canada) where biofilms were formed on the pegs. After incubation, peg lid was carefully removed from the 96‐well plates, washed twice in PBS by dipping the pegs in a 96‐well plate and then dipped in a challenge 96 well microplate containing 100 µL of different concentrations of Aβ (0 – 1 µm) or PenStrep (0 – 5 × 10^−2^ U Pen or µg mL^−1^ Strep) made in LB media. After overnight incubation in the challenge plate, peg lids were removed, and swabs of the culture were picked from the pegs and streaked onto LB agar plates. The plates were incubated overnight at 37 °C and surviving colonies were confirmed by visual inspection and counted with the software Promega Colony Counter.

### Cell Culture Experiments: Cytotoxicity, Reactive Oxygen Species, and Imaging

The neuronal (SH‐SY5Y), intestinal (Caco‐2) and gastric MKN45 cells were grown in cell culture medium, DMEM/F12 for SH‐SY5Y and RPMI for Caco‐2 and MKN45, both with 10% fetal bovine serum (FBS) and 1% of PenStrep (100 U penicillin/0.1 mg mL^−1^ streptomycin) in a tissue culture flask at 37 °C incubator (5% CO2). Immortalized Human Pericytes were grown in the pericytes medium (ScienCell). For experiments, cells were seeded (50000 cells) to a costar 24‐well cell culture plate (cytotoxicity) and 8‐well chamber slides (imaging). Both cell lines reached 70% confluency after 2 days of incubation. To obtain the disintegrated fragments of FapC (Faβ), FapC (50 µm), and Aβ (3.5 µm) were incubated for 48 h at 37 °C and filtered through centrifugal filtration (Nanosep Bio‐Inert filters 0.2 µm), 2900 g for 30 s 6 times. The isolation of Faβ was confirmed under TEM and Faβ were concentrated using Amicon Ultra‐4 centrifugal filters (30000 MWCO) at 5000 g for 5 min. Concentrated digested fragments, were adjusted to a concentration of 1 µg ml^−1^ with deionized water, using Nanodrop. To label the Faβ, Faβ were incubated with Alexa647 (1:4) for 3 h and then purified by centrifugal filtration (10000 MWCO, 5000 g for 3 min). For cytotoxicity assay, cells were incubated with 300 µL of serum‐free media, with or without Faβ (0.04 and 0.02 µm, with respect to FapC). After 24 h of incubation, the cytotoxicity was measured by propidium iodide (PI) assay. Briefly, 2 µm of PI was added to each well and incubated for an additional 1 h. The cytotoxicity was quantified by scanning the plate for PI‐positive cells with Perkin Elmer Operetta and analyzing with Harmony High‐Content Imaging and Analysis software. The cells incubated with media alone were used as negative control (0% toxicity).

For reactive oxygen species, cells were incubated with 5 µm of Dichlorodihydrofluorescein diacetate (DCFH‐DA) for 1 h. The ROS was imaged with Perkin Elmer Operetta and quantified with Harmony software. For imaging the interaction of Faβ with SH‐SY5Y and Caco‐2 cells, cells were grown in an 8‐chamber glass slide and incubated with 0.02 µm of Faβ. Cells were fixed with 4% PFA in PBS after 90 min of incubation. The fixed cells were washed with 300 µL PBS for 5 min, thrice. Cells were permeabilized by 100 µL of 0.15% Triton X‐100 in PBS for 3 min followed by 3X washing with PBS for 5 min each to remove all permeabilizing agent. Hundred microliter of Hoechst 33 342 stain (1 µg mL^−1^) was added to the cell and incubated for 10 min for nuclear staining. Again, the cells were washed thrice with PBS for 5 min each. Then, the cells were incubated with 100 µL (2 drops per mL) of ActinGreenTM 488 ReadyProbesTM reagent in the dark. After 30 min of incubation, cells were washed thrice with PBS. Fresh PBS was added to cells and confocal imaging was performed with a Leica TCS SP8 while the light panel was set for excitation emission at 488 nm for actin staining, 361/497 nm for the nucleus stain and 647 nm for Alexa 647. Images were processed and analyzed by Fiji ImageJ software. Internalization of Faβ in Caco‐2 cells was imaged via Z‐stack imaging.

### Cell Adhesion Property of FapC and CsgA Amyloids after Treatment with Aβ

FapC or CsgA (10 µm) were labelled with Alexa647 (1:4 molar ratio) and incubated with Aβ peptide (1 µm) for 48 h at 37 °C. The disintegrated fragments (10 µm for both FapC and CsgA) obtained after incubation with Aβ were added to the Caco‐2 cells grown in an 8‐well chamber glass slide, as described above and incubated for 24 h. In comparison, FapC or CsgA amyloids alone (10 µm), labelled with Alexa647, were incubated with the cells under the same conditions. To study the ability of Aβ to interact and disintegrate FapC or CsgA amyloids, attached to the cell surface, Alexa647 labelled FapC or CsgA amyloids were incubated with the Caco‐2 cells for 24 h. Later, cells were washed with fresh media to remove any un‐attached FapC or CsgA amyloids and 1 µm of Aβ dissolved in serum‐free media was added into the cells. The cells were incubated for additional 24 h. All the cells in this experiment were fixed in 4% PFA, washed with PBS and then subjected to confocal imaging as described above. The size and number of the FapC or CsgA amyloids attached to the membrane of Caco‐2 cells were quantified from the images using Fiji ImageJ software.

### Statistical Analysis

All experiments were repeated in triplicate unless specified. Data were presented as mean standard deviation (SD). For zebrafish larvae experiments, 20 larvae were injected with Aβ for MALDI experiment while 5 larvae per group were used for imaging. The significance was determined by one‐way ANOVA followed by Turkey's test and p values less than 0.05 (*) and 0.005 (**) were considered significant and highly significant, respectively. *P*‐value greater than 0.05 was considered insignificant. All data were analyzed via GraphPad Prism 9.

## Conflict of Interest

The authors declare no conflict of interest.

## Author Contributions

I.J., T.P.D. and D.E.O. conceived the project and arranged funding. S.A.A. performed ThT, DLS, ELISA, CD, SEM, anti‐microbial and antibiofilm assays, fluorescence imaging, and zebrafish experiments. K.H.K.C. performed TEM and cell culture. H.F. and A.B. performed confocal imaging and image analysis. W.P.O. synthesized microbial amyloids and performed self‐seeding ThT experiments. I.J. and A.K. analyzed the data and prepared figures. I.J., T.P.D. and D.E.O. wrote and edited the manuscript. All authors agreed on the presentation of the manuscript. Authors would like to acknowledge Prof. Matthew Chapman for providing CsgA mutant strain (MC4100).

## Supporting information

Supporting Information

## Data Availability

The data that support the findings of this study are available from the corresponding author upon reasonable request.
