# Supplementary material for: Alzheimer's Progenitor Amyloid‐β Targets and Dissolves Microbial Amyloids and Impairs Biofilm Function
Source: Adv Sci (Weinh). 2023 Aug 18;10(29):2301423. doi: 10.1002/advs.202301423 (PMC10582422; doi:10.1002/advs.202301423)
Supplement: Supplementary file 1 — Supporting Information [file ADVS-10-2301423-s001.pdf]

ADVANCED SCIENCE
Open Access

# Supporting Information

for *Adv. Sci.*, DOI 10.1002/advs.202301423

Alzheimer's Progenitor Amyloid-$\beta$ Targets and Dissolves Microbial Amyloids and Impairs Biofilm Function

*Syed Aoun Ali, Ka Hang Karen Chung, Helen Forgham, William P. Olsen, Aleksandr Kakinen, Arunpandian Balaji, Daniel E. Otzen*, Thomas Paul Davis* and Ibrahim Javed**

# Alzheimer's progenitor Amyloid-β targets and dissolves microbial amyloids and impairs biofilm function

*Syed Aoun Ali,[1] Ka Hang Karen Chung,[1] Helen Forgham,[1] William P. Olsen,[2,3] Aleksandr Kakinen,[1,4] Arunpandian Balaji,[1] Daniel E. Otzen,[2*] Thomas Paul Davis,[1*] Ibrahim Javed[1*]*

[1]Australian Institute for Bioengineering and Nanotechnology, The University of Queensland, Brisbane Qld 4072, Australia

[2]Interdisciplinary Nanoscience Center (iNANO), Aarhus University, Gustav Wieds Vej 14, 8000, Aarhus C, Denmark

[3]Sino-Danish Center (SDC), Eastern Yanqihu Campus, University of Chinese Academy of Sciences, 380 Huaibeizhuang, Huairou District, Beijing 101400, China

[4]Institute of Biotechnology, HiLIFE, University of Helsinki, Helsinki, Finland

Correspondence: Ibrahim Javed: i.javed@uq.edu.au; Thomas Paul Davis: t.davis@uq.edu.au; Daniel E. Otzen: dao@inano.au.dk

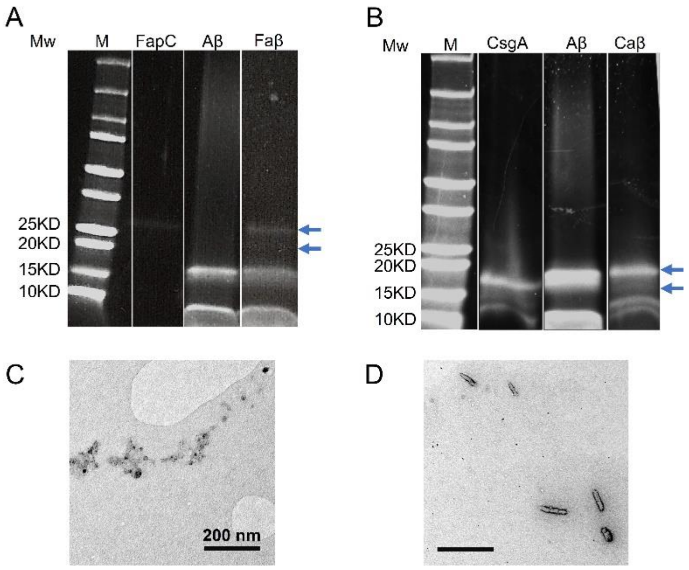


**Supplementary Figure 1.** SDS-PAGE of FapC (A) and CsgA (B) after treatment with Aβ. The bands of disintegrated FapC and CsgA, shown with arrow, were digested, subjected to protein extraction and analysed under TEM for the morphology. TEM micrographs of the disintegrated fragments of FapC (C) and CsgA (D) showed a mixture of aggregates and smaller fibrils.

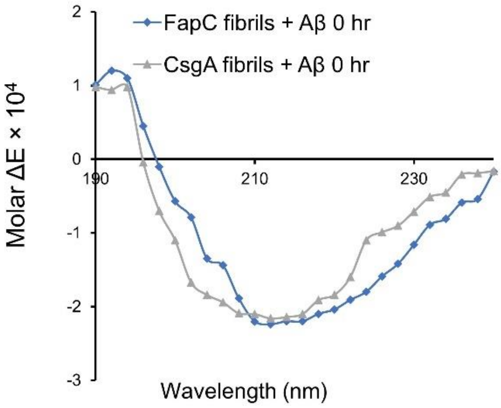


**Supplementary Figure 2.** Circular Dichroism (CD) spectroscopy of the freshly mixed Aβ monomers (3.5 µM) and FapC or CsgA fibrils (50 µM).

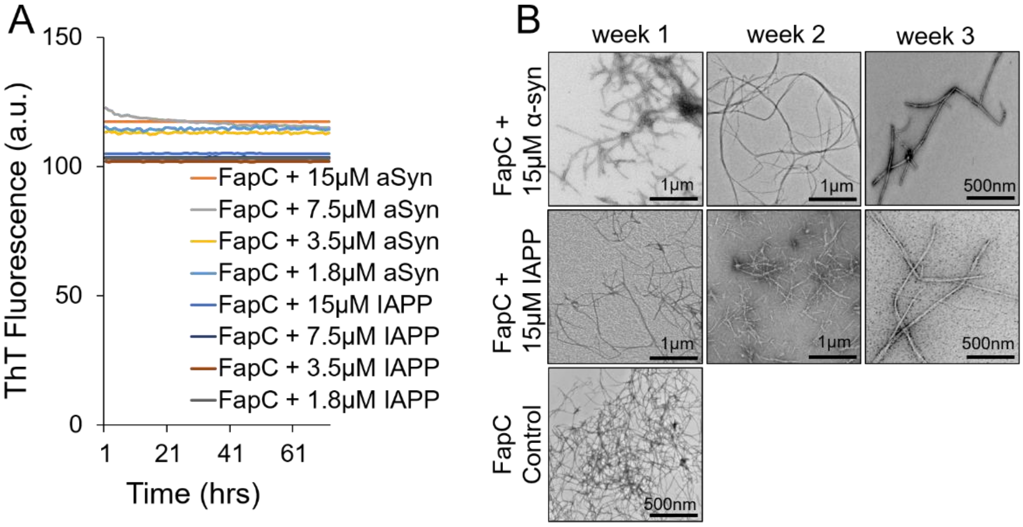


**Supplementary Figure 3.** (A) ThT assay of FapC amyloids incubated with different concentrations of human islet amyloid polypeptide (IAPP) or α-Synuclein (aSyn). (B) TEM images of FapC amyloids incubated with IAPP and aSyn.

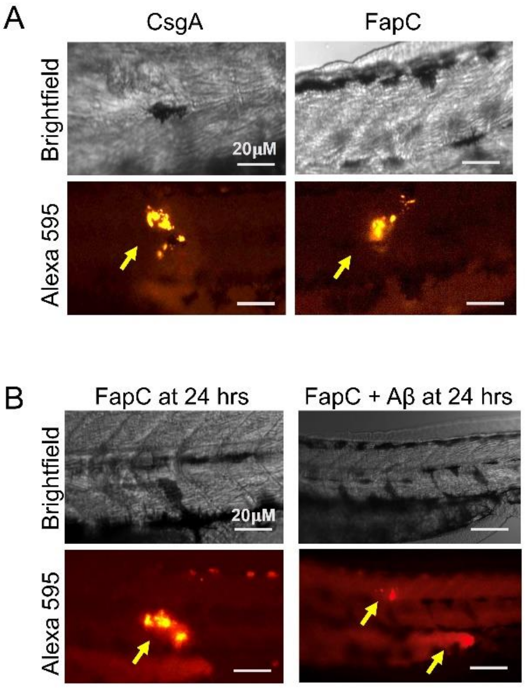


**Supplementary Figure 4.** (A) CsgA and FapC (labelled with Alexa 595) and microinjected to the tail muscle of zebrafish larvae as a control. (B) Zebrafish larvae microinjected with FapC (Alexa 595 labelled) to the tail muscle and Aβ injected to the cerebroventricular tissues showed reduction in the FapC fluorescence in the tail muscle 24 hr post microinjection, as compared to the larvae that received FapC microinjection alone.

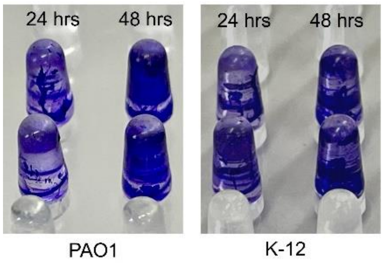


**Supplementary Figure 5.** Biofilms made by PAO1 and K12 on the Calgary Biofilm Device (CBD) for 24 and 48 hrs.

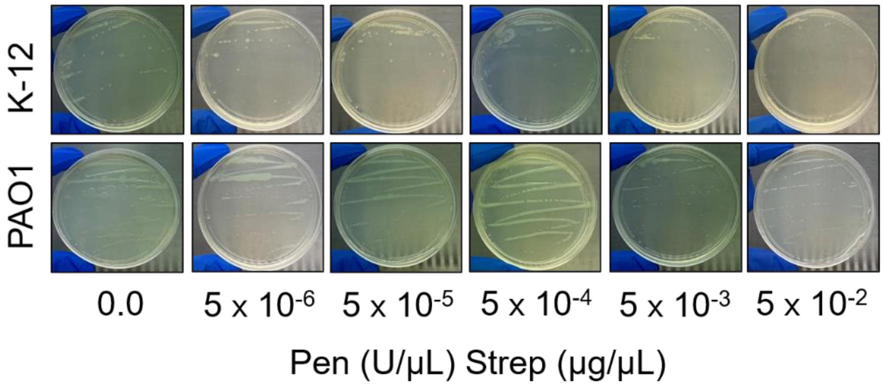


**Supplementary Figure 6.** Swab culture of biofilms made by PAO1 and K12 on the pegs of the lids, under different concentrations of Penicillin and Streptomycin antibiotics.

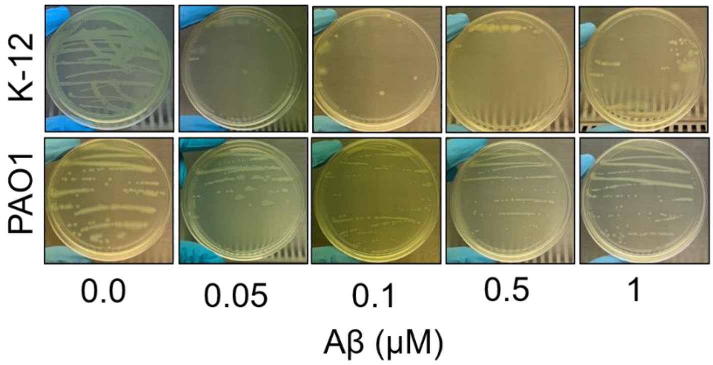


**Supplementary Figure 7.** Swab culture of biofilms made by PAO1 and K12 on the pegs of the lids, under different concentrations of Aβ.

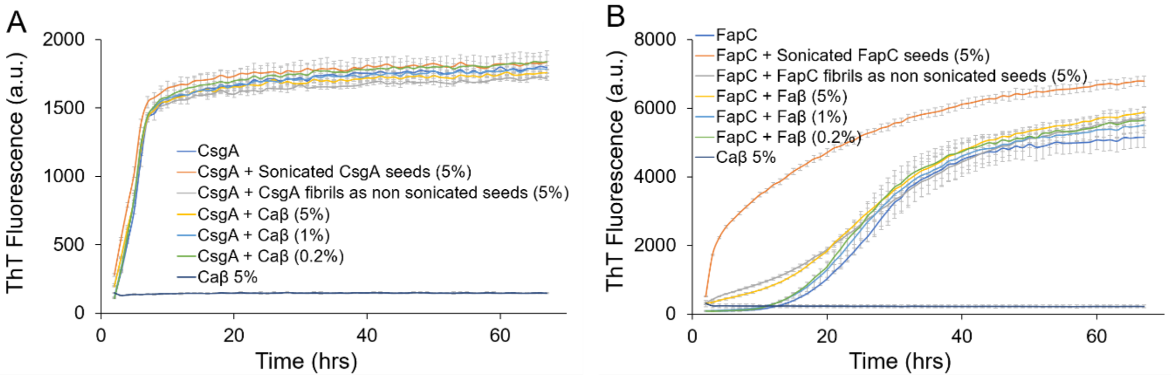


**Supplementary Figure 8.** ThT assay for CsgA (A) and FapC (B) monomers (15 µM) incubated with respective sonicated seeds, non-sonicated full-length fibrils or different concentrations (0.2-5%) of Aβ-remodelled CsgA (Caβ) or FapC (Faβ). Caβ or Faβ didn't show any seeding effect for FapC or CsgA monomers. Only, sonicated seeds of FapC were able to self-seed the fibrillization of FapC monomers.

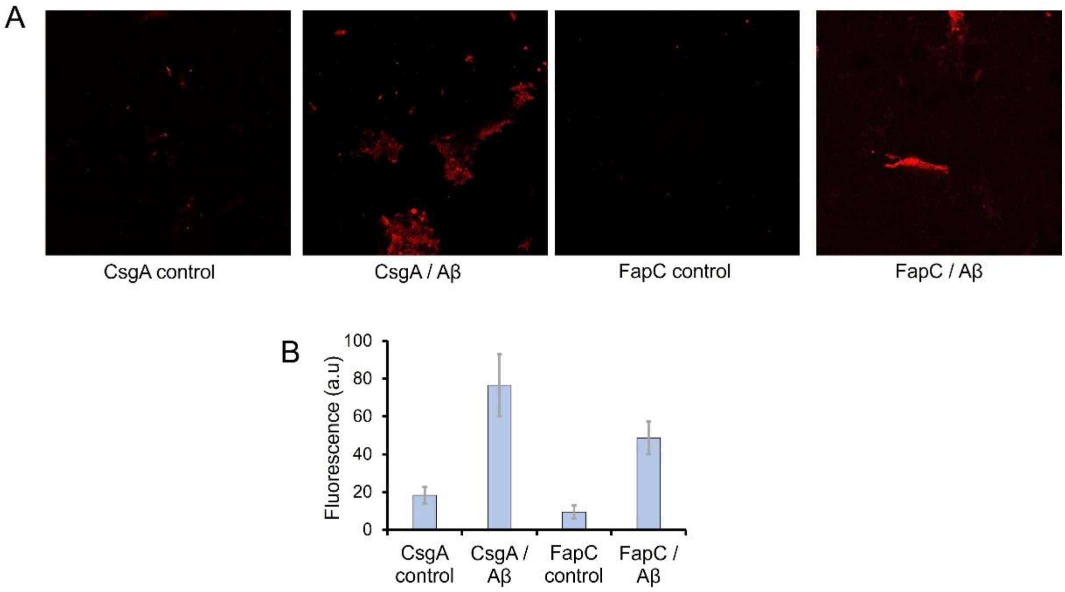


**Supplementary Figure 9.** (A) The fluorescent images and (B) quantification of fluorescence intensity *via* imageJ, in the cell culture media of Caco2 cells treated with fluorescent labelled CsgA and FapC (48 hrs) and then with Aβ (12 hrs).

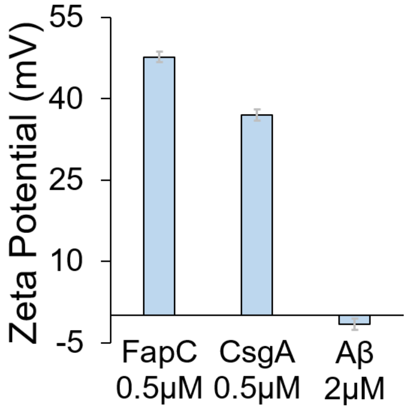


**Supplementary Figure 10.** Zeta potential of FapC or CsgA amyloids and Aβ monomers.

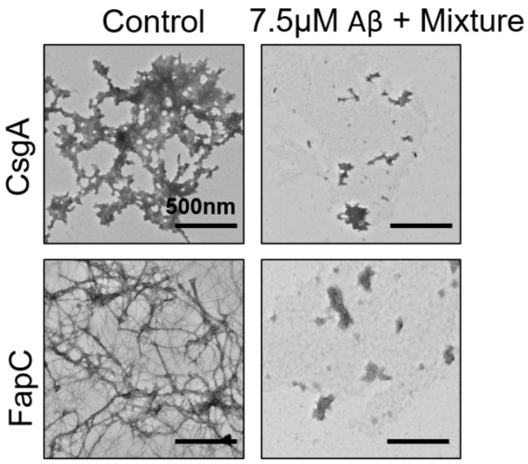


**Supplementary Figure 11.** TEM images of FapC or CsgA amyloids coated with a mixture of 5µM glucose, 5 µM cholesterol and 1µM of bovine serum albumin and then incubated with Aβ.

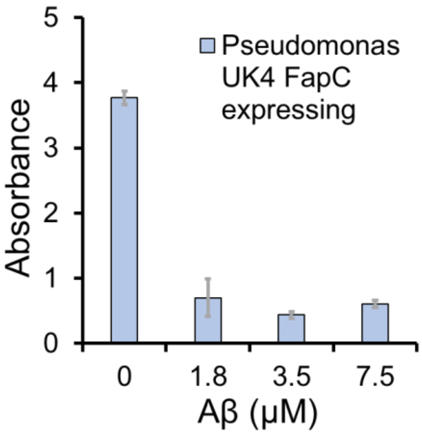


**Supplementary Figure 12.** Aβ induced disruption of biofilm made by *Pseudomonas UK4* FapC expressing strain is similar to what observed with *Pseudomonas aeruginosa PAO1*.

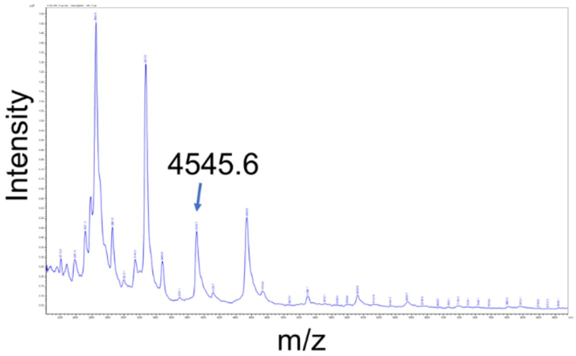


**Supplementary Figure 13.** MALDI analysis of Aβ incubated with trypsin (1% w/w) for 6 hrs.

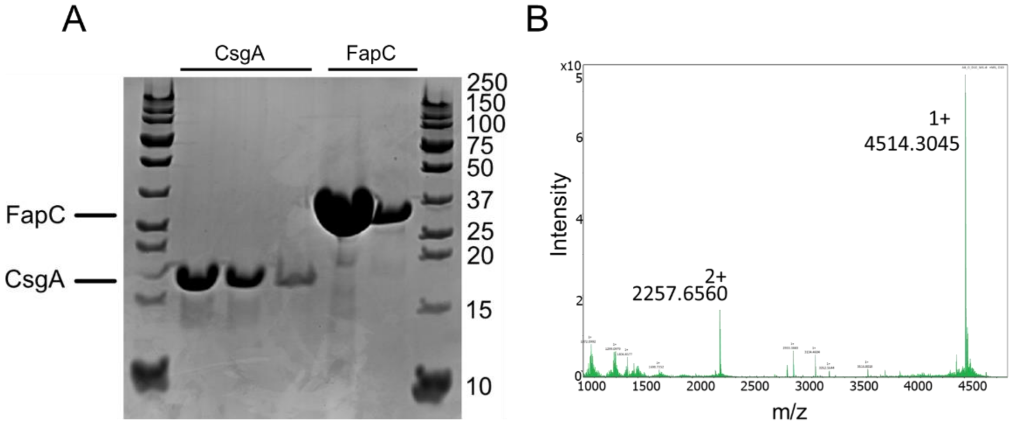


**Supplementary Figure 14.** SDS-PAGE for the purity of FapC and CsgA proteins (6 mg/mL) and MALDI analysis for the purity of Aβ peptide.
